# Catheter Displacement and Penetration of a Vaginal Cuff Following Laparoscopic Adjustable Gastric Banding: A Case Report

**DOI:** 10.7759/cureus.67497

**Published:** 2024-08-22

**Authors:** Martin Nguyen, Samuel Aulick, Torin Walters

**Affiliations:** 1 Radiology, West Virginia School of Osteopathic Medicine, Lewisburg, USA; 2 Clinical Sciences, West Virginia School of Osteopathic Medicine, Lewisburg, USA; 3 Radiology, Cabell Huntington Hospital, Huntington, USA

**Keywords:** phi angle, vaginal cuff penetration, roux-en-y gastric bypass, band migration, laparoscopic adjustable gastric banding

## Abstract

This case report describes a rare complication following laparoscopic adjustable gastric banding (LAGB) in a 47-year-old woman. The patient, who had a history of obesity and a previous hysterectomy, presented with dyspareunia. Upon examination, a catheter was visualized in the upper vaginal canal, which penetrated the right vaginal cuff and caused significant pain. Imaging revealed that a catheter from the LAGB device had penetrated the vaginal cuff. This unexpected migration of the catheter necessitated surgical intervention for removal. The case underscores the importance of monitoring for unusual symptoms in patients with a history of LAGB, as this procedure, while minimally invasive and generally safe, can have serious long-term complications. These complications may include gastric erosion, perforation, band migration, and, in this rare instance, vaginal cuff penetration. The report emphasizes the need for healthcare providers to maintain a high level of suspicion for such complications, particularly in patients presenting with atypical symptoms post LAGB. It also highlights the interdisciplinary approach required to manage these complex cases, involving both general surgery and radiology teams. To the best of our knowledge, this is one of the first cases with a history of LAGB, which was associated with the complication of penetration of the vaginal cuff.

## Introduction

It is estimated that about 42.4% of adults in the United States are obese without any significant differences between males and females [1]. Management of this condition may include behavioral, medical, and surgical options. Among these, surgery has the most reliable outcomes [2]. Laparoscopic adjustable gastric banding (LAGB) is one of the most frequently used minimally invasive interventions for morbid obesity [3,4]. LABG procedure requires the application of a silicone band around the upper part of the stomach. The band can be regulated via a subcutaneous port, which enables the injection or removal of fluid [5]. While LAGB has been shown to be a relatively safe technique with minimal perioperative complications, the re-operation rate is relatively high compared to other types of bariatric surgeries, predominantly due to gastric erosion, gastric perforation, band migration, pouch dilation, abscess formation, and slippage [4,6-9]. Band migration is a long-term complication with unclear mechanisms. Here, we present a case of LAGB presented with an unusual symptom of dyspareunia. Further investigation detected the band catheter penetrating the vaginal cuff, which necessitated a surgical intervention for catheter removal.

## Case presentation

**Figure 1:**
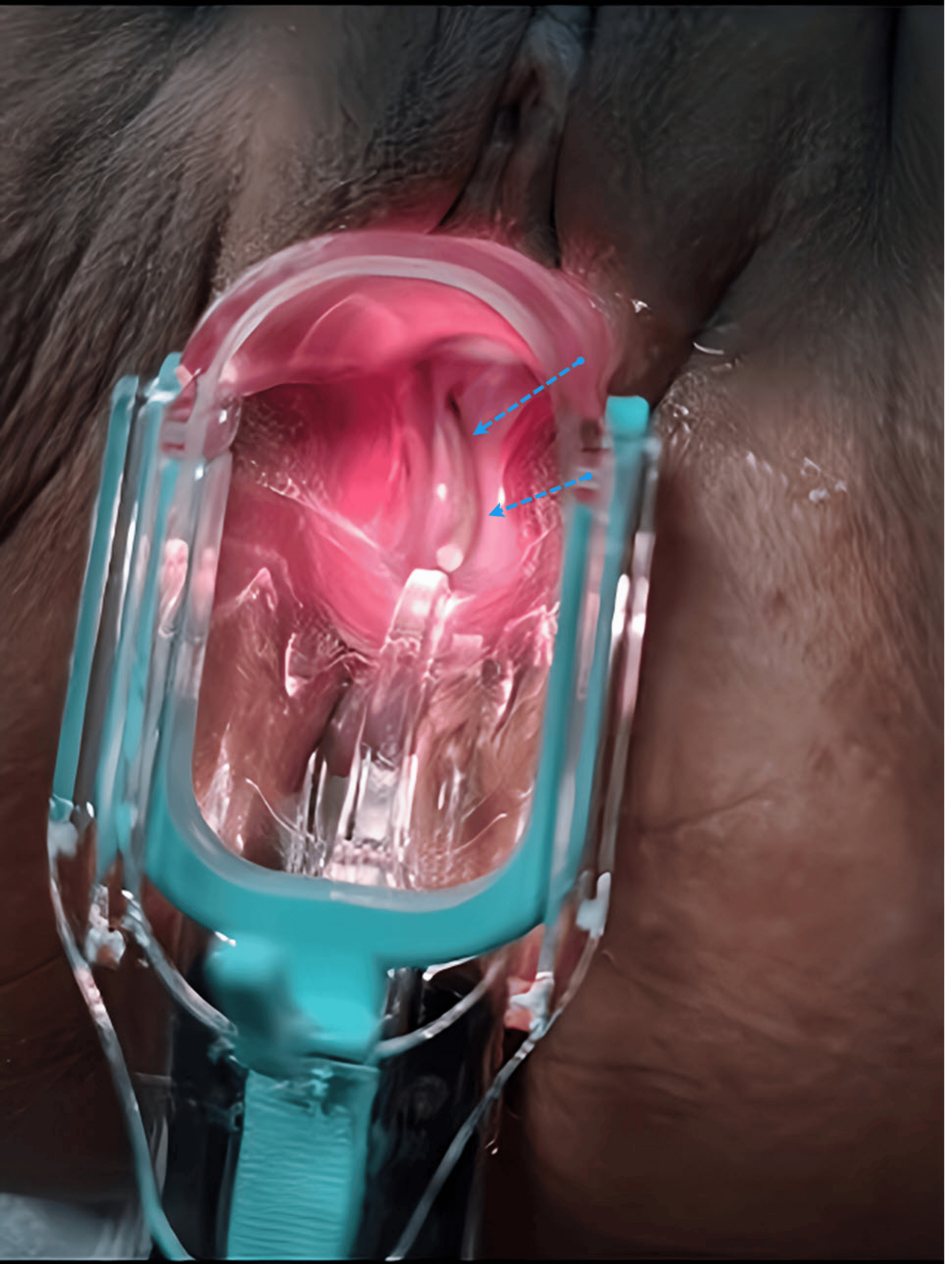
Catheter penetration of the vaginal cuff and a portion of it could be visualized through speculum examination (blue dotted arrows).

**Figure 2:**
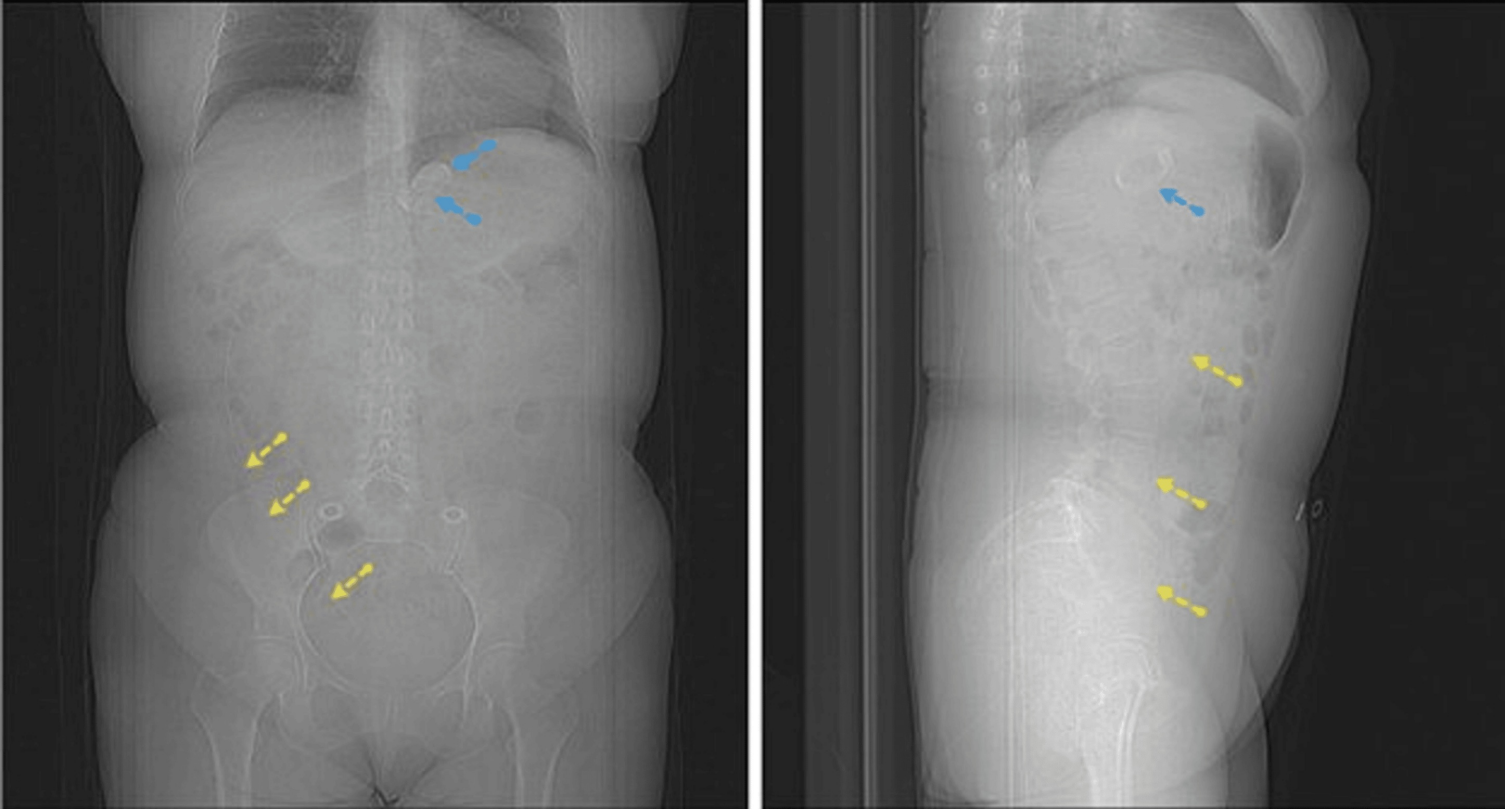
Computed tomography with contrast (left, coronal plane; right, sagittal plane). A gastric band (blue dotted arrows) and device catheter (yellow dotted arrows) were visualized. The catheter was freely suspended inside the abdominal cavity without any port attachment.

**Figure 3:**
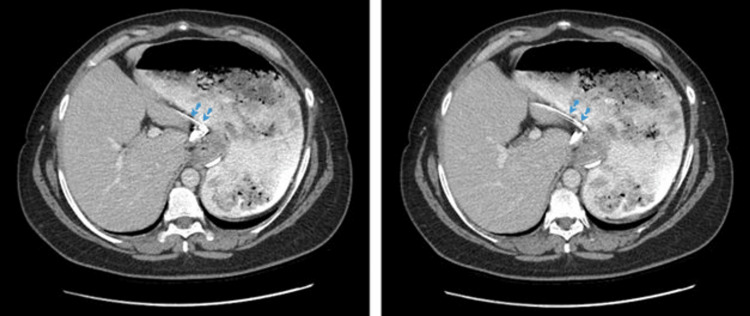
Computed tomography with contrast, axial plane. Proximal end of the catheter (blue dotted arrows).

**Figure 4:**
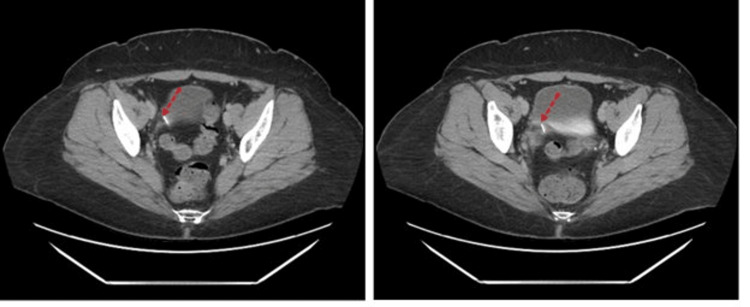
Computed tomography with contrast, axial plane. Catheter (red dotted arrows) of the gastric banding device coursing between the vagina and bladder at different levels.

**Figure 5:**
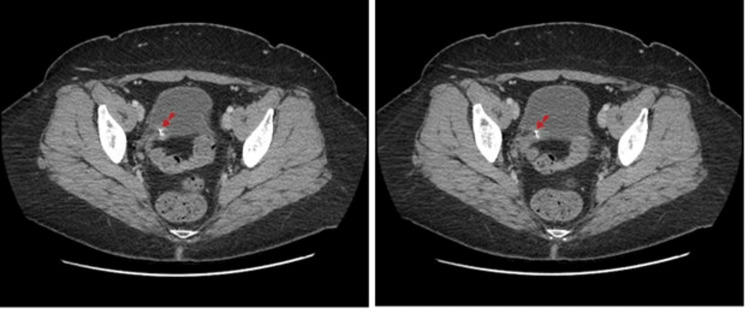
Computed tomography with contrast, axial plane. Device catheter (red dotted arrows) penetrating the vaginal cuff.

**Figure 6:**
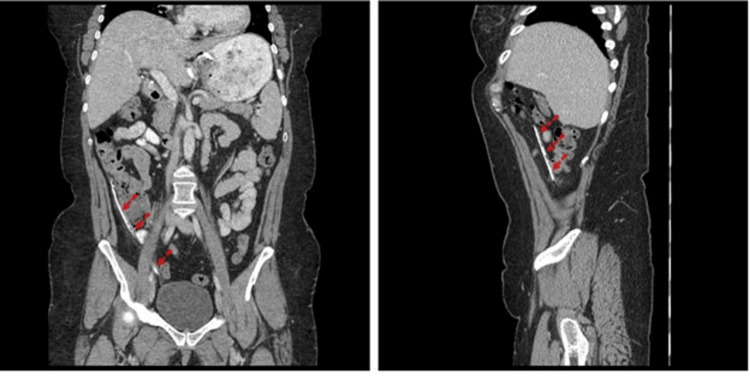
Computed tomography with contrast (left, coronal plane; right, sagittal plane). Course of the catheter in the abdominal cavity (red dotted arrows).

**Figure 7:**
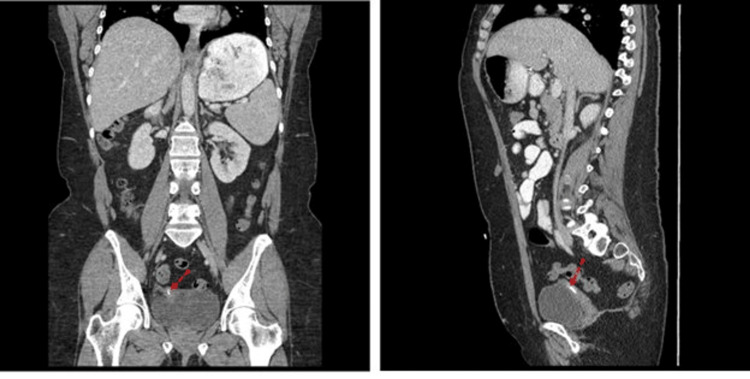
Computed tomography with contrast (left, coronal plane; right, sagittal plane). A part of the device catheter (red dotted arrows) was penetrating into the vaginal cuff and was located posterior to the bladder.

The initial presentation of the case above was a 47-year-old female who presented to the OB/GYN complaining of feeling ‘a foreign body within the vagina’ and having pain during sexual intercourse. Associated symptoms included vaginal burning, discharge, itching, and vaginal odor. Past medical history included a hysterectomy at age 30. Co-morbidities included recurrent vaginal candidiasis, bladder hyperactivity, and hypothyroidism. Prescribed medications included estradiol, levothyroxine, solifenacin, and fluticasone propionate. Prior to hysterectomy, she also had a history of gastric banding (GB) placement. Although we did not have access to the time frame of her GB procedure, she also reported that the subcutaneous port was removed after a few years. The device catheter was freely suspended in the abdomen after port removal. Her vitals were within normal limits. Body mass index (BMI) was 31.7. A speculum examination was performed, and a catheter was visualized protruding through the right vaginal cuff into the vaginal canal (Figure 1). Physical manipulation of the tube elicited vaginal as well as epigastric discomfort. Computed tomography (CT) of the abdomen and pelvis was indicated. The GB device and catheter were clearly visualized with the catheter being freely suspended inside the abdominal cavity (Figure 2). The result suggested the tube that had penetrated the vaginal cuff was the device catheter (Figures 3-7). Urinalysis was performed, and there was no evidence suggesting a urinary tract infection (UTI). The patient was later sent to the operating room, where the exposed portion of the catheter was trimmed, and the remaining portion was pulled through an incision that was made in the abdomen. The postoperative period was uneventful. She did not have any vaginal bleeding, and the pain as well as the discomfort was remarkably improved. Two days later, she was discharged and was scheduled to follow up within three months unless there were any serious complications such as infection or hemorrhage occurring.

## Discussion

For the past two decades, the prevalence of both obesity and severe obesity (SO) increased steadily among adults in the United States [1]. The prevalence of severe obesity in adults was estimated to be 9.2%, and it is more likely to occur in women than in men [1]. The age group 40-50 years of age has the highest prevalence of SO. Obesity is defined as the BMI of greater or equal to 30 kg/m^2^, while SO is defined as the BMI of greater or equal to 40 kg/m^2^ [1]. The economic burden of overweight and obesity (OAO) was estimated to be 2.19% of the global gross domestic product (GDP) ranging from 20 USD per capita in African countries to 872 USD per capita in America [10]. To put things in perspective, if we could reduce the annual OAO prevalence by 5%, that would translate into an average annual cost reduction of 429 billion USD between 2020 and 2060 [10]. Current treatment options for SO include low-calorie diets, behavioral modification, medical therapy, and surgery [11]. Dietary interventions may induce some initial weight reduction, but it is not long lasting and tends to decrease to zero after five years [12]. Behavioral and medical therapy have reported an average weight loss of 4-7 kg [13,14]. Furthermore, pharmacological agents did not appear to sustain long-term weight reduction, and weight gain tended to occur at six to nine months, which was independent of whether the treatment was continued or not [13].

**Figure 8:**
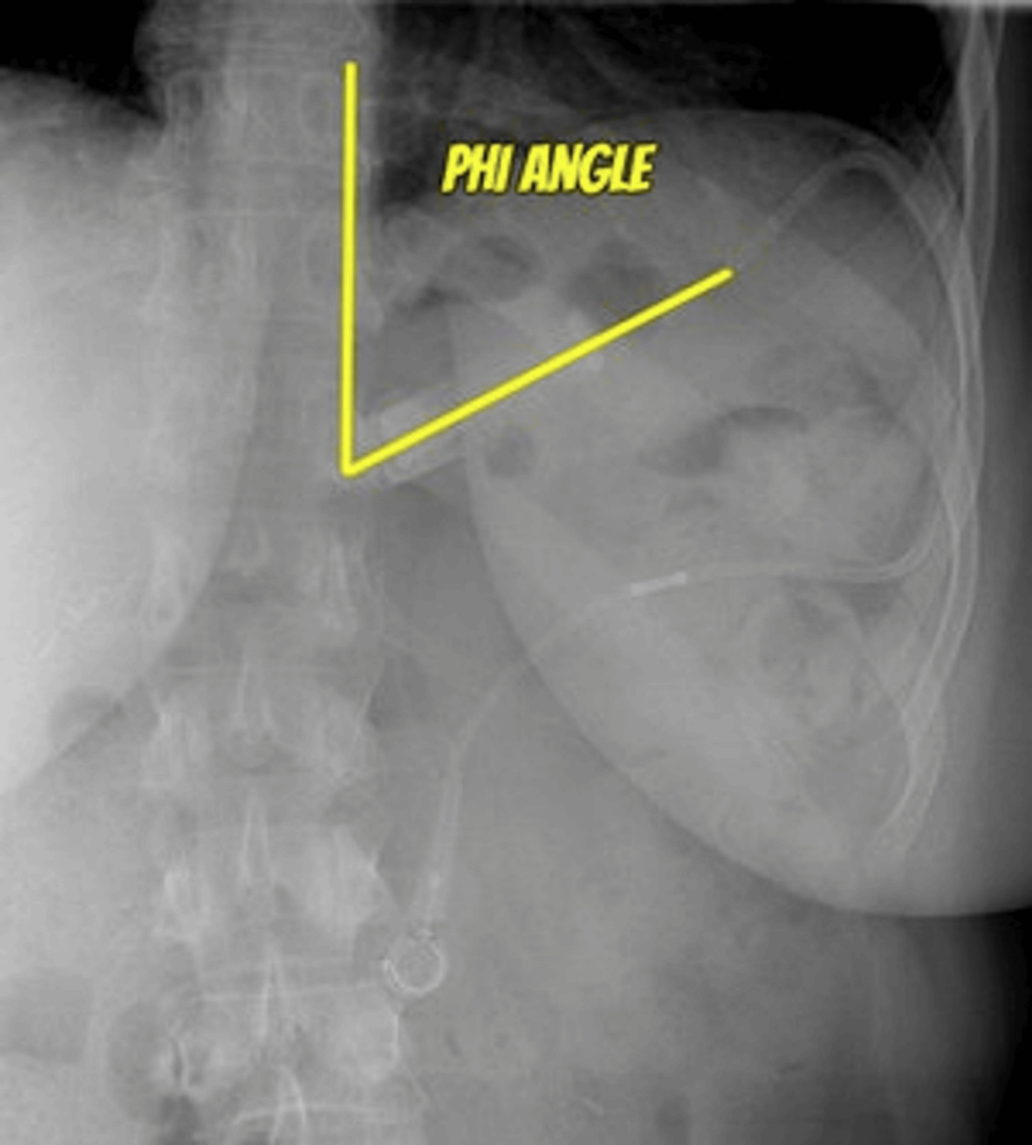
Phi angle is measured by a line parallel to the spinal column and a line parallel to the gastric band. Case courtesy of Knipe et al. [22]

Bariatric surgery has become one of the main treatment options for severe obesity due to its proven effectiveness [15,16]. Guidelines from the National Institutes of Health recommended bariatric surgery for patients with BMI > 40 kg/m^2^ or BMI > 35 kg/m^2^ who have serious co-morbidities such as severe diabetes or obstructive sleep apnea [17]. One of the least invasive interventions of bariatric surgeries is LAGB [6,18]. Since its introduction in 1993, this procedure played a significant role in the development of bariatric surgery [6]. With a minimally invasive approach, it helped encourage both physicians and patients to consider it as a potential option for the treatment of SO [6]. LAGB is one of the most popular surgical techniques in Europe, Australia, and Latin America [18]. Since its approval by the U.S. Food and Drug Administration in 2001, LAGB has been performed more commonly in the United States [19]. This procedure involves the placement of a silicone band with an inflatable balloon at the superior part of the stomach, effectively dividing it into a small gastric pouch and the remaining distal part. The area between those areas is the stoma, which is adjustable via a tubing connecting the balloon and a subcutaneous abdominal port. By injecting or removing saline from the port, the diameter of the stoma is adjusted accordingly [19]. On postoperative day one, patients will undergo fluoroscopic evaluation to rule out contrast extravasation from possible iatrogenic injury [19]. The gastric pouch should be symmetric and measure about 3-4 mm [20]. Moreover, 50 g of opaque food should be emptied from the stomach in 15-20 minutes [20]. Otherwise, the persistence of solid food for more than 30 minutes is considered delayed emptying [20]. Additionally, the phi (φ) angle is assessed. It is an angle created by a vertical line parallel to the spinal column with a line parallel to the plane of the GB [19] (Figure 8). A normal phi angle should lie 4-5 cm below the left hemidiaphragm and range from 4° to 58° [21].

Early results of LAGB were promising with low morbidity and a comparable weight loss achievement versus other procedures [23,24]. However, our understanding of its long-term complications is still limited, and a majority of current publications regarding this topic only reported the results based on 24-48 months of follow-up [6]. However, there were some papers with a significantly longer follow-up period. In 2003, Suter et al. [6] reported a study of 317 patients having LAGB with a mean duration of follow-up of 74 months. The mean BMI and the mean age were 43.5 kg/m^2^ and 38 years, respectively. About one-third (33.1%) of the patients developed late complications, the most common of which included band erosion (9.5%), a leakage in port or catheter (7.6%), and pouch dilatation/slippage (6.3%) [6]. Failure was defined as a major reoperation or an excess weight loss (EWL) of less than 25%. The latter signaled there was insufficient weight loss. The failure rates at 18 months, three years, five years, and seven years post-operative were 13.2%, 23.8%, 31.5%, and 36.9%, respectively [6]. Thus, there was a trend of increasing rates of late complications over time. The success rate (EWL > 50%) was maximal at 24 months at 53.8% and then declined progressively to 42.9% at seven years after the procedure [6]. In another study of 45 patients with a mean follow-up duration of 105 months, Camerini et al. [25] demonstrated that BMI one year after the operation was 79% of its pre-operative value (p < 0.0001). However, from there on, the BMI increased linearly over time with an average increase of 0.42 units of BMI per year [25]. Failure of weight loss is defined as BMI > 35 kg/m^2^ at five years, and this complication was observed in 34.6% of LAGB patients and in 4.2% of RYGB patients [26]. Thus, weight loss outcomes strongly favor the latter group [14,26]. Furthermore, it is currently suggested that replacement of the band after a previous failure should be discouraged [27]. In those cases of replacement of another band, the re-operative rate was as high as 45% [28].

Some authors concluded that, despite the good short-term outcomes, the constant risk of long-term complications may lessen the favorability of the LAGB procedure in the management of SO [6,25]. In 2008, Tice et. al. [14] conducted a meta-analysis to compare the effectiveness of LAGB versus Roux-en-Y gastric bypass (RYGB). It consisted of 14 comparative studies, and the results demonstrated that EWL at one year was consistently greater for RYGB (mean difference 26%, p < 0.001). Both procedures have an operative mortality rate of less than 0.5%. While LAGB has a better perioperative complication profile versus RYGB (5% vs. 9%, respectively), it also has higher reoperation rates (16% vs. 24%, respectively). Additionally, patient satisfaction was higher in the RYGB group (p = 0.006). The authors suggested that RYGB should remain the primary bariatric surgery to treat obesity in the United States [14].

Complications of LAGB may be grouped into intraoperative, early postoperative, and late postoperative categories [27]. The most common late complications include band erosion into the stomach, pouch dilatation, leakage from port-tube connection, and GB prolapse [6,19,27,27]. In our patient, the catheter has been penetrating into her vaginal wall and was detected on CT. Our current hypothesis as to how this occurred is that, once the catheter was removed from the subcutaneous port and left suspended in the abdomen, it could migrate freely. Afterward, she underwent a hysterectomy, which may have created a favorable environment for the catheter to go through the vaginal cuff. Port complication rates range from 7.3% to 23.9% [29,30]. About 20% of all the revision surgeries were due to port or tubing problems [27]. Although they are rarely life-threatening, this group may cause significant morbidity and seems to be related to the length of follow-up [27]. One of the problems in this group is tube disconnection, which may occur in 1-5% of all LAGB cases [19]. In our patient, because we could not retrieve her previous history regarding the reason why the port was temporarily removed, our hypothesis was that port removal was necessitated due to pain or infection for a course of medical treatment. However, she failed to follow up afterward. This leads to a freely suspended catheter without notice, and the patient may not be aware of its long-term potential complications, such as infection, adhesion, or perforation. Tube disconnection may be confirmed by contrast injection into the port, which will cause extravasation under fluoroscopy [27]. Regarding port infection, it may be a signal of GB erosion, and this must be ruled out by endoscopy [27]. To the best of our knowledge, this was one of the first cases with a LAGB history that is associated with vaginal pouch penetration caused by the GB device catheter.

## Conclusions

LAGB is the least invasive surgical procedure for SO. While it has been proven that LAGB has a very good profile regarding short-term complications, results worsen over time. Long-term complications are more likely to occur than RYGB. Here, we reported an atypical case with a history of LAGB presented with dyspareunia. Imaging modalities demonstrated that the catheter penetrating the vaginal cuff necessitated a revision surgery to address the problem. This underscored the importance of a high clinical suspicion in this population, as well as a multidisciplinary approach between different specialties, especially general surgery and radiology.
